# Young Infant Mortality Associated with Preterm and Small-for-Gestational-Age Births in Rural Bangladesh: A Prospective Cohort Study

**DOI:** 10.1016/j.jpeds.2024.114001

**Published:** 2024-06

**Authors:** Jennifer A. Applegate, Md Shafiqul Islam, Rasheda Khanam, Arunangshu Dutta Roy, Nabidul Haque Chowdhury, Salahuddin Ahmed, Dipak K. Mitra, Arif Mahmud, Mohammad Shahidul Islam, Samir K. Saha, Abdullah H. Baqui

**Affiliations:** 1 Department of International Health, Bloomberg School of Public Health, Johns Hopkins University, Baltimore, MD; 2 Projahnmo Research Foundation, Dhaka, Bangladesh; 3 Department of Public Health, School of Health and Life Sciences, North South University, Dhaka, Bangladesh; 4 Child Health Research Foundation, Dhaka, Bangladesh

**Keywords:** cohort study, neonatal mortality, preterm birth, second month of life, small for gestational age, small vulnerable newborn, young infant

## Abstract

**Objective:**

To assess the relative risk of mortality in infants born preterm and small for gestational age (SGA) during the first and second months of life in rural Bangladesh.

**Study design:**

We analyzed data from a cohort of pregnant women and their babies in Sylhet, Bangladesh, assembled between 2011 and 2014. Community health workers visited enrolled babies up to 10 times from birth to age 59 days. Survival status was recorded at each visit. Gestational age was estimated from mother’s reported last menstrual period. Birth weights were measured within 72 hours of delivery. SGA was defined using the INTERGROWTH-21st standard. We estimated unadjusted and adjusted hazard ratios (HRs) and corresponding 95% CIs for babies born preterm and SGA separately for the first and second month of life using bivariate and multivariable weighted Cox regression models.

**Results:**

The analysis included 17 643 singleton live birth babies. Compared with infants born at term-appropriate for gestational age, in both unadjusted and adjusted analyses, infants born preterm-SGA had the greatest risk of death in the first (HR 13.25, 95% CI 8.65-20.31; adjusted HR 12.05, 95% CI 7.82-18.57) and second month of life (HR 4.65, 95% CI 1.93-11.23; adjusted HR 4.1, 95% CI 1.66-10.15), followed by infants born preterm-appropriate for gestational age and term-SGA.

**Conclusions:**

The risk of mortality in infants born preterm and/or SGA is increased and extends through the second month of life. Appropriate interventions to prevent and manage complications caused by prematurity and SGA could improve survival during and beyond the neonatal period.

Globally, an estimated 5.2 million children die before their fifth birthday—nearly one-half of these deaths occur in the first month of life.^1^ The leading causes of neonatal mortality are complications from preterm birth (PTB), infections, and intrapartum complications. Complications from PTB are the leading causes of death in children younger than 5 years of age, accounting for about 1 million deaths worldwide.^2^ Progress to reduce PTB rates globally has been slow. Around the world, an estimated 13.4 million babies were born prematurely in 2020, with the greatest rates (13.4%) in Southern Asia.^3^, ^4^, ^5^

Although not considered a direct cause of death, intrauterine growth restriction contributes to neonatal and child mortality globally.^6^, ^7^, ^8^ Small for gestational age (SGA) often is considered a proxy for intrauterine growth restrictions and is attributed to infants “weighing below the 10th centile of sex-specific birth weight by completed gestational age of a given reference population.”^9^^,^^10^ The prevalence of SGA, and associated mortality risk, varies by the choice of standard or reference population used in the analysis.^11^^,^^12^ The INTERGROWTH-21st standards—prescriptive, international standards for fetal growth—currently are considered the most appropriate global reference population for defining SGA.^13^^,^^14^ In 2020, an estimated 23.4 million babies were born SGA worldwide. In South Asia, 40.9% of all live births were classified as SGA, accounting for nearly two-thirds (63%; 14.8 million) of the global burden.^5^ Infants born SGA are more likely to suffer from neonatal infections, perinatal respiratory depression, jaundice, polycythemia, hypoglycemia, poor feeding, and hypothermia.^7^ The increased likelihood of these morbidities contributes to a greater mortality risk for infants born SGA.^7^

Both PTB and fetal growth restriction (often estimated by SGA) are underlying pathways to low birth weight (LBW), which historically has served as an important marker for infant mortality and long-term health outcomes.^15^, ^16^, ^17^ Evidence shows considerable overlap in PTB, LBW, and SGA and, taken together, these conditions account for most of young infant deaths globally. A recent *Lancet* Series proposes a new conceptual framework where LBW, PTB, and SGA are captured under the holistic term of the “small vulnerable newborn,” which also emphasizes that newborns can belong to more than one vulnerability group.^5^^,^^15^

Although there are multiple small vulnerable newborn phenotypes, the combinations of PTB and/or SGA are of particular interest, given the global prevalence of both conditions and associated mortality risk.^5^^,^^15^ Recent analyses estimate that 1.5 million infants in 2020 were born both too soon (preterm) and too small (SGA).^5^ It is well established in the literature that being born with both conditions is associated with a high risk of mortality in the neonatal period.^5^^,^^6^^,^^18^, ^19^, ^20^, ^21^ However, few studies in low- and middle-income countries have examined the mortality risk for small vulnerable babies outside of the neonatal period, including the second month of life. The World Health Organization’s guidelines for *Integrated Management of Childhood Illness* and *Managing Possible Serious Bacterial Infections in Young Infants* both use the age cutoff of 0-59 days because the signs and management of possible serious bacterial infections for infants in the second month of life are more similar to neonates than older children (2-59 months).^22^^,^^23^ Understanding the burden and risk of mortality for small vulnerable newborns during early infancy (0-59 days) can better guide policies and interventions to improve infant survival, which will be important for reducing the under-5 mortality rate and achieving the third Sustainable Development Goal.^24^ In this study, we evaluated the relative risk of mortality associated with LBW, PTB, SGA, and different PTB and SGA phenotypes—using the INTERGROWTH-21st standards—in a cohort of young infants during the neonatal period and the second month of life in a rural area of Bangladesh.

## Methods

### Study Site, Participants, and Design

Pregnant women were enrolled as part of an observational cohort study to assess the incidence and causes of community-acquired serious infections among young infants in South Asia (Aetiology of Neonatal Infection in South Asia or ANISA), which was conducted in Bangladesh, India, and Pakistan from 2011 to 2014. This study presents only data from the site in Sylhet, Bangladesh. The study population is predominantly rural, residing in 2 subdistricts (Zakiganj and Kanaighat) of the Sylhet district, located in the northeastern part of Bangladesh. This population was chosen because of poor maternal and newborn health indicators, including high neonatal mortality.^25^ The detailed methods of the ANISA study in Bangladesh were published previously.^26^ This study involved prospective surveillance of married women of reproductive age who received visits from study-trained resident female community health workers (CHWs) every 2 months. Each frontline worker had at least a 10th-grade education and was responsible for surveillance—including identification of pregnancies, births, infant illness, and deaths—in rural communities of ∼3000 persons.^25^ At the bimonthly surveillance visits, CHWs asked married women of reproductive age about their last menstrual period (LMP), using recent important dates (eg, religious festivals, important events in the area) to improve recall, and recorded the date in their personal registers.^27^ A woman was considered pregnant if 2 months had passed since her LMP, at which time her pregnancy was registered and the woman was followed until delivery.^28^

CHWs received 6 weeks of training on maternal and newborn health and an additional 7 days of training on newborn assessment, including birth weight measurement.^27^^,^^29^ As part of training and standardization, CHWs measured 5 separate babies’ birth weights at the study area hospital, which were checked against the weight measurements taken by a training physician. Supervision of CHWs and quality assurance procedures were similar to other protocols in the study area and are described elsewhere.^27^, ^28^, ^29^, ^30^ To summarize, field supervisors directly supervised CHWs through routine supervisory visits as well as standardized exercise sessions on newborn assessment and data collection.^27^ For example, refresher training on newborn assessment and birth weight measurement was provided as needed during fortnightly supervisory meetings, or at least quarterly. Further, an electronic data management system was designed to run data consistency tests. If an inconsistency or incomplete entry was identified in the system, then the field data collection team was notified and the CHW conducted field verification to resolve the issue.^27^

Eligible participants were women with live babies whose birth weight could be measured within 3 days of birth. CHWs collected maternal, demographic, and household information at enrollment. Information on labor, delivery, birth weight, gestational age, and the newborn’s health status was collected by CHWs at the first visit after birth, ideally within 24 hours of delivery.^25^^,^^28^ Birth weights were measured by project CHWs once in the immediate postpartum period, no later than 72 hours after delivery, using a pediatric and infant digital weighing scale (Tanita BD-585; precision 10 g), which CHWs calibrated daily and documented in a register routinely checked by their supervisors.^28^^,^^29^ Gestational age was estimated from the mother’s LMP, which was collected as part of routine surveillance. Direct supervisors of CHWs randomly verified LMP of pregnant women as part of their supervisory checklist.

Babies enrolled in the study were visited at home by a CHW 3 times in the first week of life and once a week thereafter, up to 10 times from age 0-59 days.^25^^,^^26^^,^^28^ Survival status was recorded at each visit. For this analysis, we included singleton live births. We excluded infants with birth defects, missing birth weight, as well as those with missing or improbable gestational age data. Women who remained pregnant at the end of the study were considered censored in the analysis.

### Measurements

The outcomes of interest were mortality in the neonatal period (deaths <28 days) and in the second month of life (deaths 28-59 days). The exposure variables of interest included gestational age and birth weight. We categorized gestational age calculated using LMP into 6 categories. We aimed to use the World Health Organization’s definitions for categorization of preterm birth: extremely preterm (<28 weeks), very preterm (28 to <32 weeks), or moderate-to-late preterm (32-36 weeks). For babies born at term (≥37 weeks), we used the American College of Obstetricians and Gynecologists categorizations^31^: early term (37-38 weeks) and full term (≥39 weeks). We considered mortality by gestational age in weeks and found a small number of babies born <28 weeks, so decided to combine the <28 weeks and 28-31 weeks categories to represent the babies born early preterm (<32 weeks). We also found substantial variability in the outcomes for babies in the moderate-to-late preterm group (32-36 weeks), so we divided this group into 32-33 weeks and 34-36 weeks.

We considered mortality for every 500 g of birth weight and aimed to categorize LBW per the World Health Organization’s definitions: normal birth weight (≥2500 g), LBW (<2500 g), very LBW (<1500 g), and extremely LBW (<1000 g). However, we did not have any babies in our study population weighing <1000 g and found substantial variation in outcomes for infants with birth weight in the 1500 to <2500 g category. Thus, we decided to categorize birth weight by 500 g, including a 1000 to <1500 g category and a cutoff for 2000 g.

SGA was defined as birth weight less than the 10th percentile for the infant’s gestational age and sex using the INTERGROWTH-21st standards.^13^ We categorized size for gestational age into 3 categories: SGA (<3%) and SGA (3%-<10%) and appropriate for gestational age (AGA; ≥10%). We constructed 4 mutually exclusive categories combining size for gestational age (either AGA or SGA) and term or preterm birth: term-SGA, preterm-AGA, preterm-SGA, and term-AGA (reference group).

In addition, we explored household, nutritional, reproductive, and other known risk factors for infant mortality by calculating frequency distributions of categorical variables. The household wealth scores were calculated using principal component analysis based on housing materials and household possessions, and categorized into wealth quintiles.

### Statistical Analysis

We calculated prevalence of LBW, PTB, SGA, and combinations of gestational age and size for gestational age categories in our study population. We created a schematic diagram, as has been done in previous analyses,^6^^,^^7^^,^^32^^,^^33^ to show the distribution of infants born with 1 or more of these conditions (No. [%]), and labeled with different small vulnerable newborn phenotypes.^15^^,^^32^ To identify risk factors for young infant mortality, we examined unadjusted associations of neonatal mortality (<28 days) and mortality in the second month of life (28-59 days) with potential infant risk factors (gestational age, birth weight, size for gestational age, combination categories, sex), maternal risk factors (age, mid-upper arm circumference [MUAC], parity, education, religion, tobacco use, antenatal care, iron supplementation, pregnancy complication, place of delivery [ie, home or facility], skilled assistant at delivery, delivery complication), and household risk factors (wealth, paternal education, use of biomass fuel, kitchen location) using log-rank test of equality for the equality of survivor function. The percentages of missing were: maternal age (0.01%), skilled assistant at delivery (0.01%), and MUAC (6.4%). We used hot-deck imputation to impute missing values for some variables, including maternal age, skilled assistant at delivery, and MUAC.^34^

We calculated Kaplan-Meier survival estimates to compute daily risk of infant mortality during the first 2 months of life (0-59 days) and created survival curves for the following categories: gestational age, birth weight, birth weight centile, and combinations of gestational age and birth weight centile. For bivariate and multivariable regression analysis we employed weighted Cox regression to estimate unadjusted hazard ratios (HRs) and adjusted hazard ratios (aHRs) and corresponding 95% CIs to determine the relative risk of mortality in small vulnerable newborn categories (ie, preterm and/or SGA) compared with babies born at term and size AGA in the young infancy period. Before applying weighted Cox regression, we fitted the Cox proportional hazards model, and tested the proportional hazards assumption by visually inspecting the log-log survival plots and Schoenfeld residuals global test. A few covariates violated the proportional hazards assumption; thus, we fitted weighted Cox regression.^35^ Separate models were fitted for mortality in the neonatal period and the second month of life. For the neonatal model, newborns entered the analysis at birth and were followed until death, loss to follow-up, or reaching the age of 27 days. For the second month model, infants who survived the neonatal period entered the analysis at day 28 and were followed up until death, loss to follow-up, or reaching the age of 59 days. We did not include independent covariates—gestational age, birth weight, and combination categories—in the same model at the same time when calculating the aHR. Rather, we used separate weighted Cox regression models for gestational age, birth weight, and combination categories to calculate aHR while controlling for other covariates that were significant at a 20% level in the bivariate analysis.^36^ Both religion and use of biomass fuel were dropped because they were not significant in the bivariate analysis. We dropped the maternal parity and skilled assistant at delivery covariates from the adjusted models due to their high correlation with maternal age and place of delivery, respectively. Quantitative data were analyzed using Stata, version 18 (StataCorp LP)^37^ and the coxphw-4.0.3 package from R software (version 4.2.3).^38^

### Ethical Clearance

The study protocol was reviewed and approved by the institutional review board of the Johns Hopkins Bloomberg School of Public Health and the ethical review committee of the International Centre for Diarrhoeal Disease Research in Dhaka, Bangladesh.^25^

## Results

**Figure 1:**
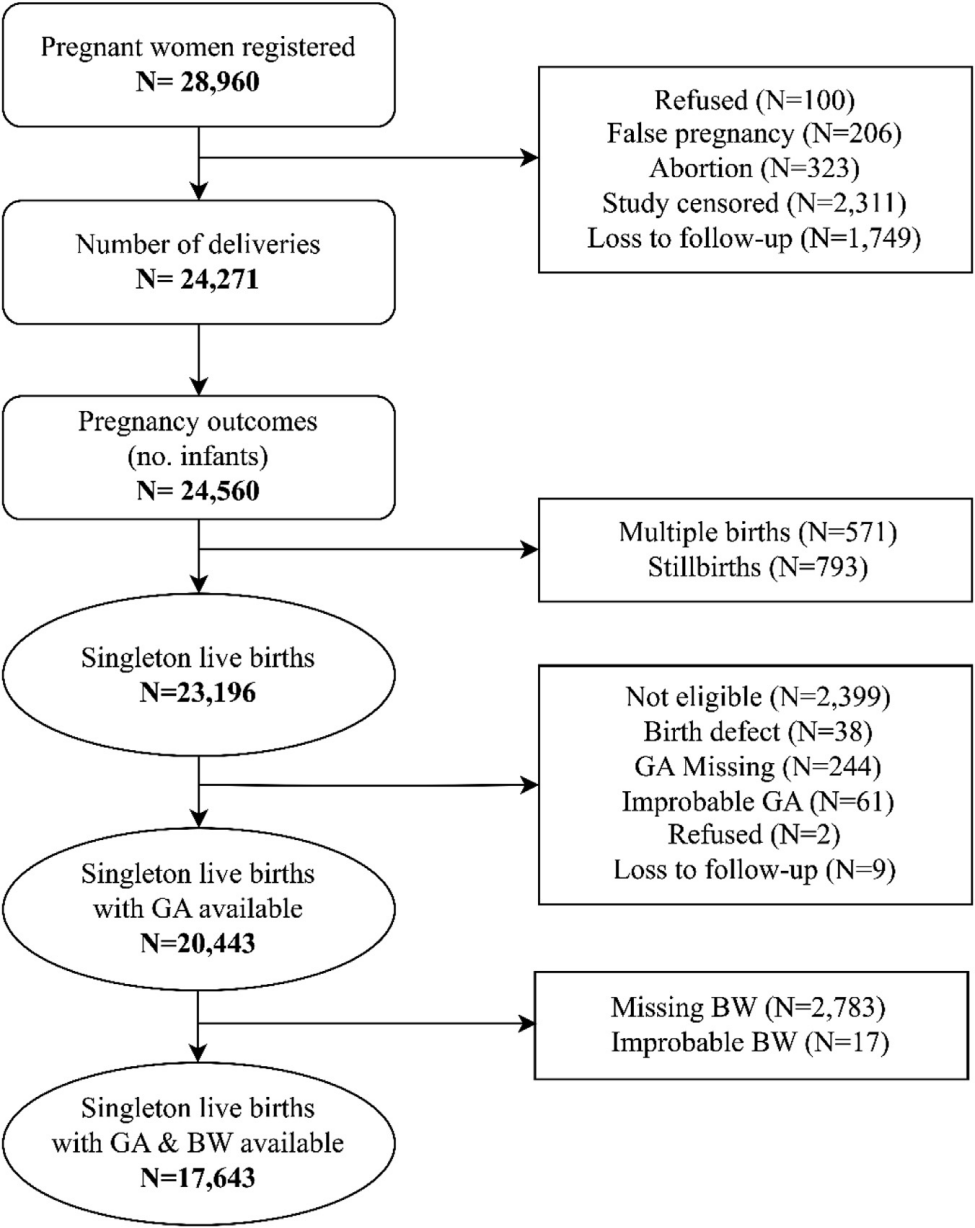
Study flow diagram including singleton live births with gestational age and birth weight available. *BW*, birth weight; *GA*, gestational age.

A total of 28 960 pregnant women were enrolled in the parent study, from whom there were 24 271 (83.8%) deliveries and 23 767 live births (including multiple births). A total of 23 196 singleton live births were eligible for inclusion in this analysis. Of these infants, gestational age was missing for 1.1% (n = 244) and birth weight was missing or improbable for 12.1% (n = 2800) of these infants. Thus, the total number of infants included in our analysis was 17 643 (Figure 1).

Table I shows baseline characteristics of our study population, including how important baseline characteristics differed between those babies who died and those that were alive at the end of follow-up, which is indicated by the *P* values provided. Across our sample, most mothers (58.5%; n = 10 316) were 20-29 years old and had some education, completing primary school (37.8%, n = 6664) or secondary school or more (37.7%, n = 6656). For 28.9% (n = 5092) of women, this was their first child. Although only 15.8% (n = 2792) of women reported tobacco consumption (ie, smoking and chewing tobacco) during pregnancy, 61.7% (n = 10 892) indicated that they had been exposed to passive or indirect smoking during their pregnancy. In total, 60.3% women (n = 10 635) reported receiving at least 1 antenatal care visit, and 90.8% (n = 16 024) reported taking iron tablets during their pregnancy. Almost all women reported delivering the baby at home (92.9%, n = 16 388), and few births were attended by a skilled birth attendant (9.2%, n = 1628). One or more complications during pregnancy and/or delivery were reported by 13.7% (n = 2423) and 16.9% (n = 2981) of mothers, respectively (Table I). Infants who died during the neonatal period (n = 370) were more likely to be male, born preterm, LBW, and SGA. Their mothers were younger, poorer, had less education, and were more likely to not have other living children. Mothers of neonates who died were more likely to report pregnancy and/or delivery complication(s) and to not have received any antenatal care. At the end of the neonatal period, 822 infants were loss to follow-up. Babies who died in the second month of life (n = 130) were also more likely to be born preterm and LBW. Their mothers were poorer, had less education, and were more likely to not have received antenatal care. Further, mothers of infants who died in the second month were more likely to report tobacco use, passive or indirect smoking, and chewing betel leaf in pregnancy (Table I).

**Table I:** Characteristics of study population, including infants who died by day 28 and 28-59 days after birth (n = 17 643)

Characteristics	<28 d			28-59 d		
	Total, No. (%)	Died, No. (%)	*P* value	Total,∗ No. (%)	Died, No. (%)	*P* value
Infant characteristics						
Gestational age, wk						
<32	438 (2.5)	54 (12.3)		366 (2.2)	9 (2.5)	
32-33	620 (3.5)	32 (5.2)	<.001	561 (3.4)	5 (0.9)	<.001
34-36	2291 (13.0)	56 (2.4)		2134 (13.0)	23 (1.1)	
37-38	3687 (20.9)	54 (1.5)		3470 (21.1)	34 (1.0)	
≥39	10 607 (60.1)	174 (1.6)		9920 (60.3)	59 (0.6)	
Total	17 643 (100)	370 (2.1)		16 451 (100)	130 (0.8)	
Birth weight, g						
1000-<1500	134 (0.8)	52 (38.8)		75 (0.5)	4 (5.3)	
1500-<2000	670 (3.8)	80 (11.9)	<.001	550 (3.3)	14 (2.5)	<.001
2000-<2500	4068 (23.1)	104 (2.6)		3760 (22.9)	46 (1.2)	
≥2500	12 771 (72.4)	134 (1.0)		12 066 (73.3)	66 (0.5)	
Total	17 643 (100)	370 (2.1)		16 451 (100)	130 (0.8)	
Size for gestational age, %						
<3	4573 (25.9)	145 (3.2)		4165 (25.3)	41 (1.0)	
3%-<10	3098 (17.6)	63 (2.0)	<.001	2902 (17.6)	28 (1.0)	.062
≥10	9972 (56.5)	162 (1.6)		9384 (57.0)	61 (0.7)	
Total	17 643 (100)	370 (2.1)		16 451 (100)	130 (0.8)	
Four newborn types						
Term-AGA	6970 (39.5)	54 (0.8)		6622 (40.3)	30 (0.5)	
Term-SGA	7324 (41.5)	174 (2.4)	<.001	6768 (41.1)	63 (0.9)	<.001
Preterm-SGA	347 (2.0)	34 (9.8)		299 (1.8)	6 (2.0)	
Preterm-AGA	3002 (17.0)	108 (3.6)		2762 (16.8)	31 (1.1)	
Total	17 643 (100)	370 (2.1)		16 451 (100)	130 (0.8)	
Sex of the baby						
Male	8879 (50.3)	212 (2.4)		8275 (50.3)	70 (0.8)	
Female	8764 (49.7)	158 (1.8)	.007	8176 (49.7)	60 (0.7)	.425
Total	17 643 (100)	370 (2.1)		16 451 (100)	130 (0.8)	
Maternal characteristics						
Maternal age, y						
<20	1726 (9.8)	49 (2.8)		1600 (9.7)	11 (0.7)	
20-29	10 316 (58.5)	194 (1.9)	.021	9627 (58.5)	66 (0.7)	.1
≥30	5601 (31.7)	127 (2.3)		5224 (31.8)	53 (1.0)	
Total	17 643 (100)	370 (2.1)		16 451 (100)	130 (0.8)	
MUAC, cm						
<22	4041 (22.9)	96 (2.4)		3752 (22.8)	39 (1.0)	
≥22	13 602 (77.1)	274 (2.0)	.161	12 699 (77.2)	91 (0.7)	.049
Total	17 643 (100)	370 (2.1)		16 451 (100)	130 (0.8)	
Parity						
0	5092 (28.9)	169 (3.3)		4680 (28.4)	35 (0.7)	
1	3901 (22.1)	53 (1.4)	<.001	3657 (22.2)	26 (0.7)	.127
2-3	5338 (30.3)	80 (1.5)		5028 (30.6)	34 (0.7)	
>3	3312 (18.8)	68 (2.1)		3086 (18.8)	35 (1.1)	
Total	17 643 (100)	370 (2.1)		16 451 (100)	130 (0.8)	
Maternal education						
None	4323 (24.5)	111 (2.6)		4032 (24.5)	58 (1.4)	
Primary	6664 (37.8)	149 (2.2)	.003	6199 (37.7)	46 (0.7)	<.001
Secondary or higher	6656 (37.7)	110 (1.7)		6220 (37.8)	26 (0.4)	
Total	17 643 (100)	370 (2.1)		16 451 (100)	130 (0.8)	
Tobacco consumption during pregnancy						
No	14 851 (84.2)	302 (2.0)		13 846 (84.2)	89 (0.6)	
Yes	2792 (15.8)	68 (2.4)	.177	2605 (15.8)	41 (1.6)	<.001
Total	17 643 (100)	370 (2.1)		16 451 (100)	130 (0.8)	
Passive or indirect smoking during pregnancy						
No	6751 (38.3)	127 (1.9)		6301 (38.3)	28 (0.4)	
Yes	10 892 (61.7)	243 (2.2)	.118	10 150 (61.7)	102 (1.0)	<.001
Total	17 643 (100)	370 (2.1)		16 451 (100)	130 (0.8)	
Chewing betel leaf during pregnancy						
No	7714 (43.7)	155 (2.0)		7177 (43.6)	32 (0.4)	
Yes	9929 (56.3)	215 (2.2)	.475	9274 (56.4)	98 (1.1)	<.001
Total	17 643 (100)	370 (2.1)		16 451 (100)	130 (0.8)	
Any antenatal care						
No	7008 (39.7)	166 (2.4)		6510 (39.6)	68 (1.0)	
Yes	10 635 (60.3)	204 (1.9)	.042	9941 (60.4)	62 (0.6)	.003
Total	17 643 (100)	370 (2.1)		16 451 (100)	130 (0.8)	
Taking iron tablet during pregnancy						
No	1619 (9.2)	53 (3.3)		1449 (8.8)	11 (0.8)	
Yes	16 024 (90.8)	317 (2.0)	.001	15 002 (91.2)	119 (0.8)	.944
Total	17 643 (100)	370 (2.1)		16 451 (100)	130 (0.8)	
Complication during pregnancy						
Yes	2423 (13.7)	76 (3.1)		2265 (13.8)	19 (0.8)	
No	15 220 (86.3)	294 (1.9)	<.001	14 186 (86.2)	111 (0.8)	.787
Total	17 643 (100)	370 (2.1)		16 451 (100)	130 (0.8)	
Place of delivery						
Home	16 388 (92.9)	341 (2.1)		15 278 (92.9)	125 (0.8)	
Facility	1255 (7.1)	29 (2.3)	.587	1173 (7.1)	5 (0.4)	.148
Total	17 643 (100)	370 (2.1)		16 451 (100)	130 (0.8)	
Skilled assistant at delivery						
Skilled birth attendant	1628 (9.2)	36 (2.2)		1524 (9.3)	6 (0.4)	
TBA	14 596 (82.7)	306 (2.1)	.907	13 611 (82.7)	114 (0.8)	.182
Untrained	1419 (8.0)	28 (2.0)		1316 (8.0)	10 (0.8)	
Total	17 643 (100)	370 (2.1)		16 451 (100)	130 (0.8)	
Delivery complication						
Yes	2981 (16.9)	98 (3.3)		2788 (16.9)	24 (0.9)	
No	14 662 (83.1)	272 (1.9)	<.001	13 663 (83.1)	106 (0.8)	.663
Total	17 643 (100)	370 (2.1)		16 451 (100)	130 (0.8)	
Household characteristics						
Household wealth quintiles						
1 (poorest)	3576 (20.3)	91 (2.5)		3335 (20.3)	39 (1.2)	
2	3665 (20.8)	98 (2.7)	<.001	3389 (20.6)	35 (1.0)	.002
3	3716 (21.1)	86 (2.3)		3469 (21.1)	27 (0.8)	
4	3318 (18.8)	51 (1.5)		3098 (18.8)	13 (0.4)	
5 (wealthiest)	3368 (19.1)	44 (1.3)		3160 (19.2)	16 (0.5)	
Total	17 643 (100)	370 (2.1)		16 451 (100)	130 (0.8)	
Paternal education						
None	6379 (36.2)	138 (2.2)		5964 (36.3)	70 (1.2)	
Primary	6230 (35.3)	150 (2.4)	.015	5782 (35.1)	43 (0.7)	<.001
Secondary or higher	5034 (28.5)	82 (1.6)		4705 (28.6)	17 (0.4)	
Total	17 643 (100)	370 (2.1)		16 451 (100)	130 (0.8)	
Place used in the house for cooking						
Separate building (structure)†	2965 (16.8)	51 (1.7)		2769 (16.8)	14 (0.5)	
Others	14 678 (83.2)	319 (2.2)	.12	13 682 (83.2)	116 (0.8)	.064
Total	17 643 (100)	370 (2.1)		16 451 (100)	130 (0.8)	

*TBA*, traditional birth attendant.

∗ In total, 822 infants loss to follow-up in the neonatal period.

† In a separate building used as kitchen + outdoors.

**Figure 2:**
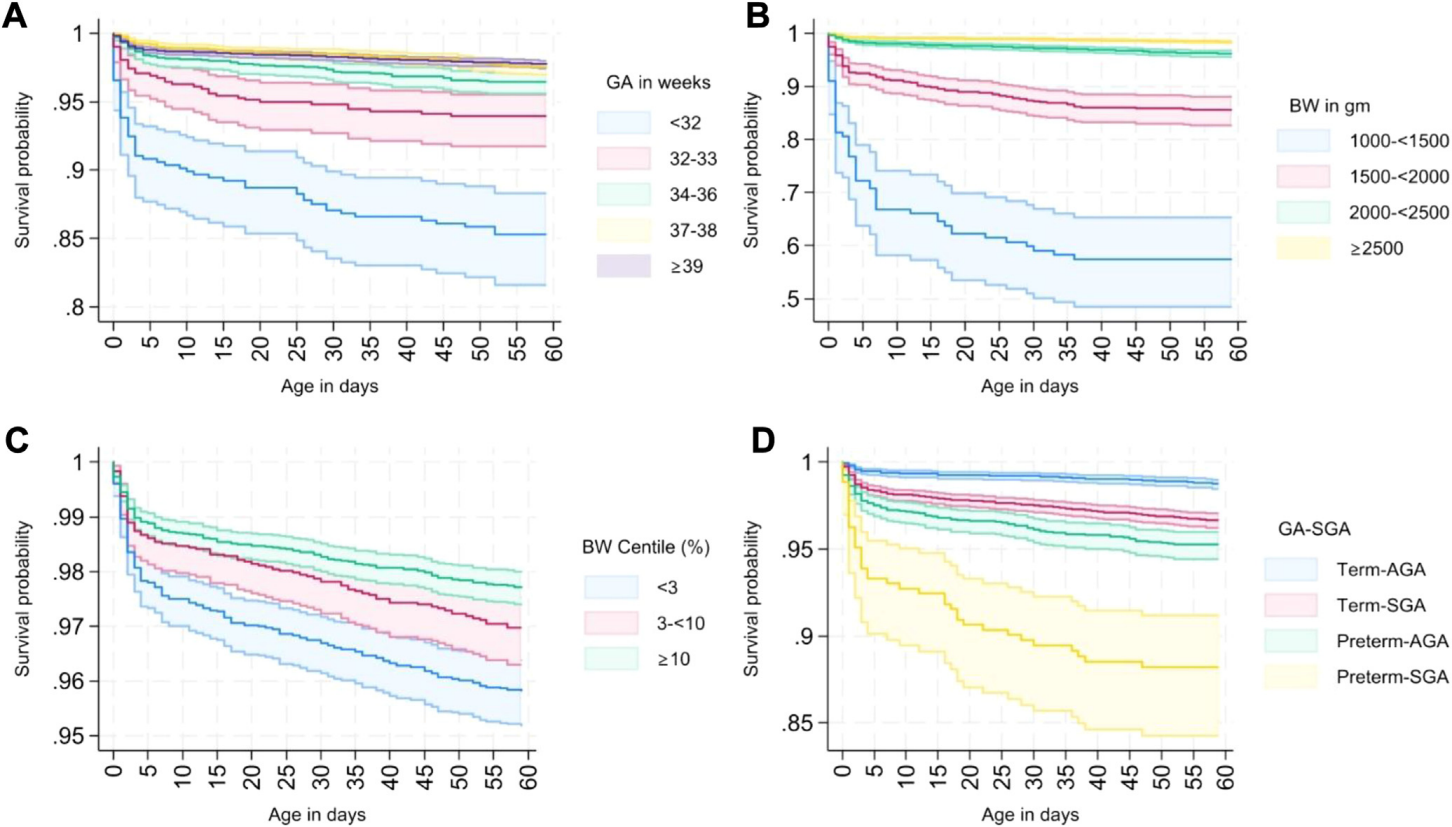
Kaplan-Meier survival estimates (95% CI) for young infants (0-59 days) by **A,** GA; **B,** BW; **C,** BW centile; and **D,** categories of 4 newborn types based on GA and size for gestational age.

Over the 2-month period of follow-up, 500 babies died—the neonatal mortality rate was 21.18 per 1000 live births (n = 370 deaths) and the mortality rate in the second month of life (28-59 days) was 7.95 per 1000 live births (n = 130 deaths) (Table II). When plotted on separate Kaplan-Meier survival curves (Figure 2), we found that probability of survival at any time point throughout the first and second months of life was lowest for babies born early preterm (<32 weeks; Figure 2, A), very LBW (<1500 g, Figure 2, B), and in the lowest BW centile (SGA [<3%], Figure 2, C). When considering babies born preterm and/or SGA, we found the probability of survival was lowest for infants born preterm-SGA, followed by preterm-AGA, and then term-SGA (Figure 2, D).

**Table II:** Risk of neonatal and second-month mortality stratified by gestational age, birth weight, size for gestation, and small vulnerable newborn types (reference: term-AGA)

	Neonatal mortality (<28 d)							Second month mortality (28-59 d)						
	Infants, No.	Deaths, No.	MR/1000 live births	HR (95% CI)	*P* value	aHR (95% CI)	*P* value	Infants,∗ No.	Deaths, No.	MR/1000 live births	HR (95% CI)	*P* value	aHR (95% CI)	*P* value
Totals	17 643	370	21.18					16 451	130	7.95				
Gestational age, wk														
<32	438	54	124.49	8.8 (6.03-12.85)	<.001	8.0 (5.47-11.69)	<.001	366	9	22.23	2.6 (1.25-5.44)	.011	2.2 (1.04-4.67)	.039
32-33	620	32	52.08	3.47 (2.24-5.4)	<.001	3.38 (2.18-5.24)	<.001	561	5	8.57	0.92 (0.36-2.36)	.862	0.74 (0.28-1.94)	.545
34-36	2291	56	24.7	1.68 (1.15-2.43)	.007	1.61 (1.11-2.34)	.013	2134	23	10.71	1.12 (0.66-1.9)	.682	1.01 (0.59-1.73)	.965
37-38	3687	54	14.81	Ref		Ref		3470	34	10.03	Ref		Ref	
≥39	10 607	174	16.56	1.1 (0.81-1.5)	0.529	1.1 (0.81-1.49)	.546	9920	59	6.01	0.61 (0.4-0.93)	.023	0.62 (0.4-0.94)	.025
Birth weight, g														
1000-<1500 g	134	52	393.24	46.85 (34.14-64.3)	<0.001	40.09 (28.6-56.2)	<.001	75	4	32.82	10.61 (3.81-29.57)	<.001	9.05 (3.2-25.61)	<.001
1500-<2000 g	670	80	121.38	12.1 (9.16-15.97)	<0.001	11.52 (8.64-15.38)	<.001	550	14	22.84	4.85 (2.71-8.66)	<.001	4.06 (2.24-7.38)	<.001
2000-<2500 g	4068	104	25.87	2.45 (1.89-3.17)	<0.001	2.42 (1.86-3.15)	<.001	3760	46	12.27	2.32 (1.59-3.39)	<.001	2.13 (1.43-3.17)	<.001
≥2500 g	12 771	134	10.58	Ref		Ref		12 066	66	5.58	Ref		Ref	
Size for gestation														
<3%	4573	145	32.09	1.97 (1.57-2.46)	<.001	1.84 (1.46-2.32)	<.001	4165	41	9.8	1.51 (1.01-2.25)	.044	1.41 (0.93-2.13)	.108
3%-<10%	3098	63	20.54	1.25 (0.93-1.67)	.136	1.22 (0.91-1.63)	.189	2902	28	9.73	1.52 (0.97-2.37)	.069	1.53 (0.98-2.39)	.063
≥10%	9972	162	16.39	Ref		Ref		9384	61	6.56	Ref			
Four newborn types														
Term-AGA	6970	54	7.82	Ref		Ref		6622	30	4.64	Ref		Ref	
Term-SGA	7324	174	24.01	3.04 (2.24-4.13)	<.001	2.85 (2.09-3.88)	<.001	6768	63	9.36	2.09 (1.35-3.25)	.001	1.91 (1.22-2.98)	.004
Preterm-SGA	347	34	99.39	13.25 (8.65-20.31)	<.001	12.05 (7.82-18.57)	<.001	299	6	18.55	4.65 (1.93-11.23)	.001	4.1 (1.66-10.15)	.002
Preterm-AGA	3002	108	36.3	4.62 (3.33-6.41)	<.001	4.33 (3.12-6.01)	<.001	2762	31	11.03	2.55 (1.53-4.22)	<.001	2.11 (1.26-3.53)	.004

*MR*, mortality rate; *Ref*, reference category.

∗ In total, 822 infants loss to follow-up in the neonatal period.

Infants were born preterm at 19.0% (3349/17643): 2.5% (n = 438) were early preterm (<32 weeks), 3.5% (n = 620) were moderately preterm (32-33 weeks), and 13.0% (n = 2291) were late preterm (34-36 weeks). Of the babies who were born at term, 20.9% (n = 3687) were categorized as early term (37-38 weeks) and 60.1% (n = 10 607) were full term (≥39 weeks) (Table I). In the first month of life, being born preterm was associated with an increase in mortality compared with babies born at term (37-38 weeks, reference group) for all preterm categories in both the unadjusted and adjusted models: early preterm (unadjusted HR 8.8, 95% CI 6.03-12.85; aHR 8.0, 95% CI 5.47-11.69); moderately preterm (HR 3.47, 95% CI 2.24-5.4; aHR 3.38, 95% CI 2.18-5.24); and late preterm (HR 1.68, 95% CI 1.15-2.43; aHR 1.61, 95% CI 1.11-2.34) (Table II). Although the magnitude declined, the increased risk of mortality persisted in the second month of life for infants born early preterm (HR 2.6, 95% CI 1.25-5.44; aHR 2.2, 95% CI 1.04-4.67). We found no difference in neonatal mortality for babies born at term. However, there was a decreased risk of mortality in the second month of life for babies born full term (≥39 weeks) compared with those born early term (HR 0.61, 95% CI 0.4-0.93; aHR 0.62, 95% CI 0.4-0.94).

More than 27% (27.6%, 4872/17643) of the infants in the cohort were born LBW (<2500 g). Compared with babies born at a normal birth weight (≥2500 g), being born LBW was associated with an increased risk of mortality in both the first and second month of life across all LBW categories. Few babies (0.8%, n = 134) were born very LBW (1000-<1500 g), but these babies had a high relative risk of mortality in the first month (HR 46.85, 95% CI 34.14-64.3; aHR 40.09, 95% CI 28.6-56.2) and second month of life (HR 10.61, 95% CI 3.81-29.57; aHR 9.05, 95% CI 3.2-25.61) compared with babies with normal birth weight (Table II).

In total, 43.5% (7671/17 643) of infants were born SGA—25.9% (n = 4573) of infants were in the <3 centile and the remaining 17.6% (n = 3098) of infants were classified in the 3 to <10 centile. Newborns classified as SGA (<3%) had an increased risk of mortality in the first month of life (HR 1.97, 95% CI 1.57-2.46; aHR 1.84, 95% CI 1.46-2.32) compared with babies who were AGA. In the second month of life, infants who were SGA (<3%) had an elevated risk of mortality in the unadjusted model (HR 1.51, 95% CI 1.01-2.25), but this difference was not significant in the adjusted model (aHR 1.41, 95% CI 0.93-2.13) (Table II).

Of the 4 newborn types, most infants were born term-SGA (41.5%, n = 7324) followed by term-AGA (39.5%, n = 6970), preterm-AGA (17.0%, n = 3002), and preterm-SGA (2.0%, n = 347) (Table I). Compared with infants born term-AGA, infants born preterm-SGA had the greatest risk of neonatal mortality (HR 13.25; 95% CI 8.65-20.31), followed by infants born preterm-AGA (HR 4.62; 95% CI 3.33-6.41) and term-SGA (HR 3.04; 95% CI 2.24-4.13) (Table II). After adjusting for covariates, a similar pattern of increased risk of neonatal mortality remained for infants born preterm-SGA (aHR 12.05; 95% CI 7.82-18.57), preterm-AGA (aHR 4.33; 95% CI 3.12-6.01), and term-SGA (aHR 2.85; 95% CI 2.09-3.88) compared with infants born term-AGA (Table II). In both unadjusted and adjusted models, infants born preterm-SGA had the greatest risk of death in the second month of life (HR 4.65, 95% CI 1.93-11.23; aHR 4.1, 95% CI 1.66-10.15), followed by infants who were preterm-AGA (HR 2.55, 95% CI 1.53-4.22; aHR 2.11, 95% CI 1.26-3.53), and term-SGA (HR 2.09, 95% CI 1.35-3.25; aHR 1.91, 95% CI 1.22-2.98) compared with infants who were term-AGA (Table II).

**Figure 3:**
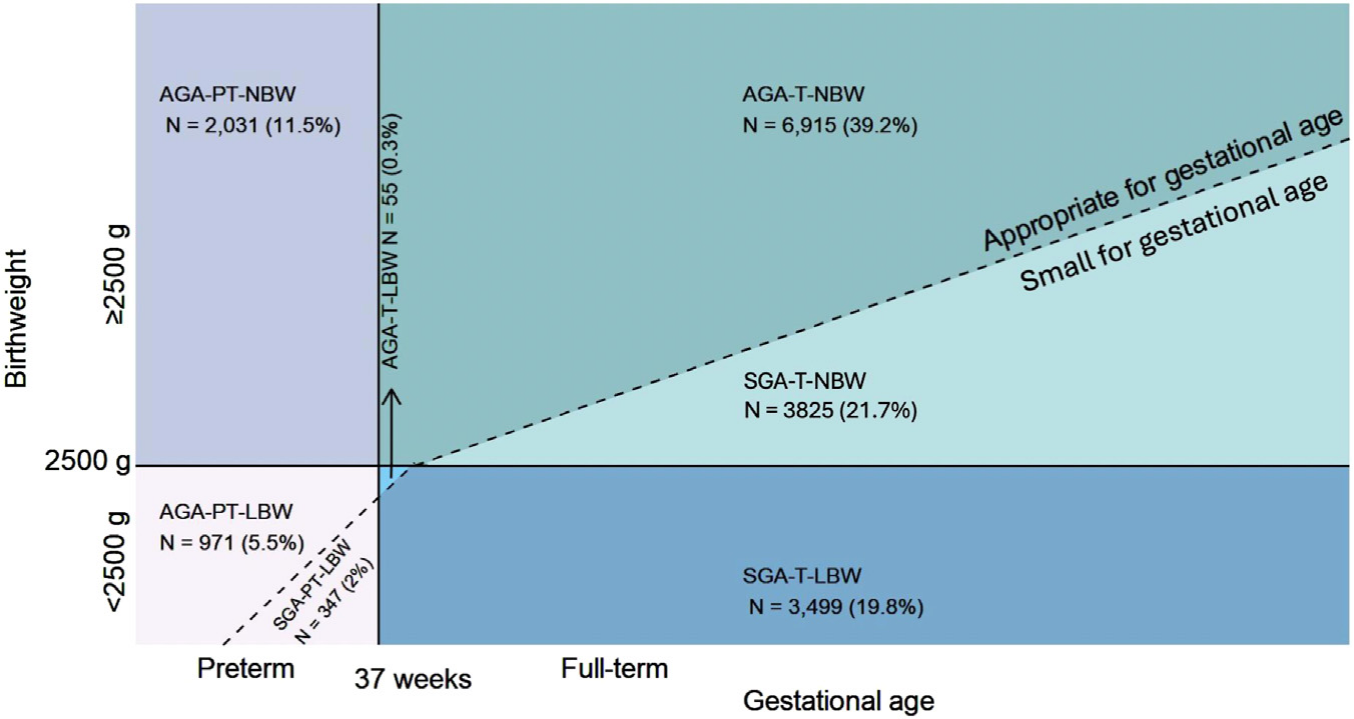
Relationship of birth weight and gestational age including different types of small vulnerable newborns in our sample (n = 17 643). The surface areas representing the numbers and percentages of phenotypic categories in the diagram were not drawn to scale. *NBW*, normal birth weight; *PT*, preterm; *T*, term.

Overall, we found that 60.8% (10 728/17 643) of the babies in our cohort had 1 or more of the conditions (LBW, PTB, SGA) and that these infants contributed to 83.4% (417/500) of the deaths; 85.4% (316/370) in the first month and 77.7% (101/130) in the second month of life. More than one-quarter of the babies were LBW (27.6%; 4872/17643), of whom 71.8% (n = 3499) were term-SGA, 19.9% (n = 971) were preterm-AGA, and 7.1% (n = 347) were preterm-SGA (Figure 3). More than two-thirds of the babies who were preterm-AGA were born at normal birth weight (67.7%, 2031/3002). Of babies born term-SGA, just more than one-half (52.2%, 3825/7324) were normal birth weight. All of the babies born preterm-SGA (n = 347) were LBW.

## Discussion

We aimed to assess the relative risk of mortality for small vulnerable newborns compared with babies born at term and AGA in the first and second months of life in a large cohort of infants followed prospectively until 59 days of age in rural Bangladesh. We found a high incidence of SGA (43.5%), LBW (27.6%), and preterm birth (19.0%) in the study population. Taken together, 60.8% of the babies in our sample had at least 1 of these conditions, which would qualify them as small vulnerable newborns. Few infants were born preterm and SGA (2%), but these infants had a nearly 12-fold increased risk of death in the first month of life and 4-fold increased risk of death in the second month of life compared with babies who were term-AGA. Babies who were preterm-AGA (17%) had more than a 4-fold increased risk of neonatal mortality and twice the risk of death in the second month of life compared with babies who were term-AGA. Babies born at term who were SGA had a smaller increased risk of mortality in the first and second month of life compared with babies born at term and appropriate size but made up a large proportion of our sample (41.5%).

The high proportion of babies born preterm in this cohort is consistent with estimates from a recent systematic review, which found that Bangladesh has one of the greatest numbers of preterm births globally.^3^ Complications of preterm birth remain a leading cause of death in newborns in Bangladesh.^39^ All preterm birth categories had greater relative risks of neonatal mortality compared with term babies—the risk was substantially greater for babies born early preterm. In the second month, only those infants born early preterm (<32 weeks) had a greater risk of death than babies born at term. The variation in survival by gestational age in our study reinforces the value of viewing preterm birth as a continuum, particularly for the management of neonates born preterm. These findings are consistent with other analyses demonstrating increasing neonatal mortality risk with decreasing gestational age.^6^^,^^30^^,^^40^ Although we did not identify any other studies that specifically quantified mortality risk for infants born preterm in the second month of life, we identified several studies that found increased risks of morbidities and mortality for infants born preterm persist throughout infancy.^3^^,^^14^^,^^18^^,^^41^, ^42^, ^43^

We found a high incidence of SGA in this population, which is consistent with other analyses that included data from South Asia.^5^^,^^7^ In this study, we used the INTERGROWTH-21st standards to classify infants born SGA vs AGA. However, there is debate about using one universal growth standard or customized fetal growth standards for specific contexts.^7^^,^^13^^,^^44^ The argument for using population-specific growth standards is to better account for the “genetic potential for growth” as well as patterns in maternal health and nutrition in pregnancy. However, it is expected that the INTERGROWTH-21st standards are currently the most appropriate standard for low- and middle-income country populations and there is less variation in fetal growth across these populations when mother’s nutritional and health needs are met.^7^^,^^13^^,^^14^^,^^45^ Looking at the various cutoffs for SGA, we found that babies born in the <3 centile had nearly twice the risk of neonatal death compared with babies who were AGA. Although the relative risk of mortality is not as great as preterm, more than one-quarter of our sample (25.9%) were categorized SGA (<3%) suggesting that a high proportion of babies in our study population are at risk of deaths as well as developmental and growth delays, late-onset infections, and respiratory and cardiovascular diseases later in life.^7^^,^^46^, ^47^, ^48^ Targeting mothers with nutritional interventions to improve fetal growth later in pregnancy, particularly weight gain, has the potential to lead to large reductions in SGA in this population.^49^^,^^50^

Most of the infants in our cohort would be considered small vulnerable newborns (60.8%, n = 10 728), and more than three-quarters of the deaths were among vulnerable infants in both the first (85.4%, n = 316) and second (77.7%, n = 101) months of life. Our findings contribute to the evidence on small vulnerable newborns in resource-limited settings, including quantifying the relative risk of mortality associated with combinations of preterm birth and SGA, and the probability of survival through the first 2 months of life. Our findings are consistent with other studies that have found a small percentage of babies were born preterm and SGA, but those who were had a substantially increased risk of neonatal mortality, compared with either condition alone, which was followed by infants born preterm and AGA, then term and SGA.^5^^,^^6^^,^^14^^,^^33^^,^^40^ We found that a similar pattern holds for mortality in the second month of life, but the magnitude of risk is lower in all categories. Our findings on variations of risk across different cutoffs of gestational age, size for gestational age, and birth weight also serve as an example of the complexities of using multiple criteria when assessing mortality in babies born too small and too soon, including the risk of missing 1 or more types of vulnerable babies in these estimates, which underscores the potential value of the more inclusive small vulnerable newborn concept.^5^^,^^15^

Historically, the Sylhet division of Bangladesh is high-burden, low-performing in terms of maternal, newborn, and child health indicators. When compared with the rest of the country, mothers in Sylhet were the least likely to receive antenatal care, had the lowest proportion of births in facilities (52%), and the second lowest proportion of births attended by a skilled provider (59.2%).^51^ Previous research in our study population in Sylhet demonstrated that facility delivery of women with intrapartum complications can reduce perinatal mortality. Still, mothers report low care-seeking for antepartum, intrapartum, and postpartum complications as the result of barriers to access (eg, economic, geographic) and poor quality of care.^52^, ^53^, ^54^ The availability of facility-based neonatal care is even more limited in the study area. The subdistrict hospital (Upazila Health Complex; catchment area ∼250 000 persons) is the closest public-sector option for inpatient management of sick young infants in our study area. However, it is not adequately equipped to manage critically ill neonates. Further, mothers in Sylhet report low care-seeking from this facility due to inconsistent availability of services (eg, lack of availability of drugs) financial constraints (eg, high user fees, transportation costs), and poor quality of care (eg, long wait times, previous experiences with disrespectful care, distrust in hospital doctors).^55^ There is an urgent need to address both supply- and demand-side barriers to improve the availability, accessibility, and quality of facility-based care for reducing perinatal mortality due to intrapartum complications and managing sick young infants.

In settings such as ours, it is imperative to sustain and scale up community-based delivery of maternal and newborn health services as well as develop integrated community-facility strategies to improve equitable access to specialized services. Evidence-based interventions delivered as part of antenatal care packages—including nutritional supplementation and screening and treatment of maternal infections—have the potential to prevent small vulnerable newborn births.^49^ A recent multicountry study, including Bangladesh, showed that among women in low-resource countries who were at risk for early preterm birth, the use of dexamethasone resulted in significantly lower risks of neonatal death.^56^ As part of postnatal care, delivering simple, appropriate interventions closer to the community to manage complications due to preterm birth—such as kangaroo mother care and treatment of neonatal infections—have been shown to improve newborn survival, but coverage remains suboptimal.^57^, ^58^, ^59^ Management of very small and sick newborns in newborn intensive care units of hospitals has been found to improve survival, but issues of access and quality persist in low-resource settings.^60^, ^61^, ^62^, ^63^ A recent study in Sylhet region of Bangladesh assessed the effect of integrating a community-facility intervention model, which included inputs to improve the quality of neonatal care at the first-level referral facility and used the existing CHW surveillance system in the study area to identify and refer sick newborns. Investigators found that the integrated model resulted in early identification and timely referral of severely ill newborns; increased care-seeking; improved treatment; and overall reductions in case fatality and neonatal mortality.^61^ In addition to improving the quality of facility-based care for neonates, greater investment is needed in community-facility strategies to improve timely identification and referral of severely ill babies to upgraded facilities. Our findings suggest that these gains in survival for small, vulnerable newborns may extend beyond the neonatal period.

A strength of this study is that it is from a large, community-based, prospective cohort where infants were identified and visited early in the neonatal period and followed throughout the second month of life. Gaps in available and high-quality data on babies born too small and too soon remain a major challenge in understanding the true burden of preterm and SGA and the associated mortality risk for small, vulnerable infants, especially in low-income countries. For example, a recent systematic review and modeling study of the global burden of preterm birth using 2014 data noted that the included data on preterm birth were disproportionately from high-income countries.^3^^,^^64^ Our study contributes evidence on mortality risk of preterm birth and SGA in a high-burden, low-resource setting that can help to fill these data gaps. Further, few studies have examined the risk of mortality associated with these outcomes throughout infancy, and our study uniquely focused on the carryover risk of mortality in the second month of life when infants remain especially vulnerable.

Our analysis had several limitations for estimating gestational age, LBW, and SGA. The use of LMP to estimate gestational age of infants has been shown to be less accurate than other methods (eg, ultrasonography) and is subject to recall bias, especially when recorded for women presenting for antenatal care later in their pregnancy or after delivery. Further, the accuracy of mothers’ recall has been found to be less precise in populations with low literacy and socioeconomic status.^29^^,^^65^^,^^66^ A recent multicountry cohort study found that when compared with early ultrasonography, LMP dating tended to underestimate gestational age for babies falling into the lower gestational age categories.^29^ For our analysis, this suggests possible misclassification of babies in lower gestational age categories (eg, early preterm). However, since we conducted bimonthly home visits to identify pregnancies in the study area, the recall duration for LMP was short, and therefore, we expect misclassification to be minimal. Further, in a later community-based study in the same population, we assessed gestational age in a birth cohort with early pregnancy ultrasound dating—1 in 8 infants was born too soon (<37 weeks). This corroborates a high burden of preterm birth, although the prevalence was lower than estimates with LMP-based dating.^67^

For our analysis on birth weight and size for gestation, a key limitation was that 2800 (12.1%) infants were excluded due to missing or improbable birth weight data. The availability and quality of birth weight data are common challenges in Sub-Saharan Africa and South Asia, especially for community-based studies where a high number of births occur at home.^16^ Of the 912 young infant deaths in our study sample, more than 45% (n = 411) had missing birth weight. Our supplemental analyses indicated that infants with missing birth weight died earlier than those babies with birth weight available, were more often born preterm, and had a greater risk of death in the first month of life. In our study sample, more than 80% of the births occur at home. However, despite the high proportion (90%) of newborns registered by the CHW in the first week of life, 43% of the neonatal deaths occurred before the CHW could reach the newborn, suggesting that some birth weight was not recorded due to an early neonatal death.^25^ Those infants missing birth weight were excluded from this analysis, which suggests that the incidence of preterm birth, LBW, and SGA—and the associated relative risk of mortality—may be underestimated in this study, particularly for babies born preterm.

In summary, our findings suggest that the relative risk of mortality associated with being born preterm and/or SGA is increased compared with babies who are born term-AGA during the neonatal period and extends through the second month of life. Thus, targeting mothers and infants with appropriate interventions to prevent and manage complications due to prematurity and SGA could have an effect on survival during and outside of the neonatal period. Future research on the effect of interventions for small vulnerable newborns should also assess the effect on survival for these infants in the second month of life.

## CRediT authorship contribution statement

**Jennifer A. Applegate:** Formal analysis, Investigation, Writing – original draft, Writing – review & editing. **Md Shafiqul Islam:** Data curation, Formal analysis, Writing – review & editing. **Rasheda Khanam:** Formal analysis, Investigation, Writing – review & editing. **Arunangshu Dutta Roy:** Data curation, Investigation, Supervision, Writing – review & editing. **Nabidul Haque Chowdhury:** Data curation, Writing – review & editing. **Salahuddin Ahmed:** Conceptualization, Data curation, Methodology, Supervision, Writing – review & editing. **Dipak K. Mitra:** Data curation, Investigation, Supervision, Writing – review & editing. **Arif Mahmud:** Investigation, Resources, Supervision, Writing – review & editing. **Mohammad Shahidul Islam:** Conceptualization, Data curation, Methodology, Supervision, Writing – review & editing. **Samir K. Saha:** Conceptualization, Funding acquisition, Supervision, Writing – review & editing. **Abdullah H. Baqui:** Conceptualization, Formal analysis, Funding acquisition, Methodology, Resources, Supervision, Writing – review & editing.

## Declaration of Competing Interest

The study was funded by the 10.13039/100000865Bill & Melinda Gates Foundation (grant no. OPPGH5307). The authors do not have a commercial or other association that might pose a conflict of interest in this research.

## Supplementary Data

Applegate_STROBE_checklist
